# Optimizing Positron Emission Tomography-Computed Tomography (PET-CT) Use in Colorectal Cancer: Identifying Patients at High Risk of Recurrence

**DOI:** 10.7759/cureus.83230

**Published:** 2025-04-30

**Authors:** Takaaki Fujimoto, Koji Tamura, Kinuko Nagayoshi, Yusuke Mizuuchi, Ayaka Ikeda, Yuichi Tanaka, Naoki Ikenaga, Kohei Nakata, Kenoki Ohuchida, Masafumi Nakamura

**Affiliations:** 1 Department of Surgery and Oncology, Graduate School of Medical Sciences, Kyushu University, Fukuoka, JPN

**Keywords:** colorectal cancer, pet-ct scan, recurrence prediction, standardized uptake value (suv max), tumor marker

## Abstract

Introduction

This study aimed to evaluate the prognostic significance of preoperative positron emission tomography-computed tomography (PET-CT) in resectable colorectal cancer (CRC), specifically examining the association between standardized uptake value max (SUVmax) and recurrence risk, stratified by pathological stage.

Methods

A retrospective analysis was conducted on 164 CRC patients who underwent PET-CT before curative resection. SUVmax values were assessed for their correlation with overall survival (OS) and recurrence-free survival (RFS). Receiver operating characteristic (ROC) curve analysis was used to determine the SUVmax cutoff for recurrence prediction. Multivariate Cox regression identified independent prognostic factors.

Results

In stage 3 CRC, SUVmax was significantly associated with recurrence risk, with a cutoff of 13.5 (area under the ROC curve (AUC) = 0.72, *p* = 0.0201), but not in stage 1 or 2 (AUC = 0.54). SUVmax was higher in patients with elevated preoperative carcinoembryonic antigen (CEA) (*p* = 0.0311), advanced T stage (*p* < 0.0001), and pathological stage (*p* < 0.0001). In multivariate analysis, high SUVmax (*p* = 0.0491) and anastomotic leakage (*p* = 0.0281) were independent predictors of worse OS, while high SUVmax (*p* = 0.0201), American Society of Anesthesiologists physical status (ASA-PS) ≥3 (*p* = 0.0049), and high blood loss (*p* = 0.0413) were associated with poor RFS in patients with stage 3 CRC. Patients with both high SUVmax and elevated postoperative CEA had significantly worse OS (*p* = 0.0352) and RFS (*p* = 0.0075).

Conclusions

Preoperative PET-CT has prognostic value in stage 3 CRC but offers limited utility in earlier stages. Given its cost and restricted predictive capacity, PET-CT should be selectively used for high-risk patients.

## Introduction

Colorectal cancer (CRC) is a malignancy with a rising incidence in developed countries, including Japan, and recurrence remains a major determinant of patient prognosis [1,2]. Accurate preoperative assessment of recurrence risk is crucial for optimizing individualized treatment strategies. Although advancements in surgical techniques and imaging modalities have improved recurrence risk prediction [3-6], the optimal combination of these methods remains uncertain.

Standardized uptake value max (SUVmax) derived from positron emission tomography-computed tomography (PET-CT) reflects tumor metabolic activity and has been recognized as a prognostic marker in various malignancies [7,8]. In CRC, elevated SUVmax has been linked to an increased risk of recurrence and metastasis [9], with studies suggesting its utility in assessing lymph node involvement [10] and correlating with tumor proliferation and recurrence risk [9]. However, the prognostic value of SUVmax alone is limited, particularly considering cost-effectiveness concerns.

Tumor markers such as carcinoembryonic antigen (CEA) and carbohydrate antigen 19-9 (CA19-9) are widely used for CRC diagnosis and monitoring and have established roles in detecting early recurrence [11,12]. These markers are routinely measured in the preoperative setting and are easily obtainable. Previous studies have identified elevated CEA and CA19-9 levels as independent prognostic factors in CRC.

In parallel, advancements in machine learning and radiomics have enabled more sophisticated analyses of PET-CT images, offering potential refinements in recurrence prediction models [13]. SUVmax, which reflects tumor metabolic activity, has also been investigated as a potential imaging-based prognostic marker. By integrating SUVmax with routinely available tumor markers such as CEA and CA19-9, the accuracy of recurrence risk stratification may be improved.

This study aims to evaluate the clinical utility of combining SUVmax with tumor markers to improve recurrence prediction in CRC patients.

## Materials and methods

Patient demographics and data acquisition

This retrospective study was approved by the Ethics Review Committee of Kyushu University Hospital (approval number: 23336). In accordance with the Ethical Guidelines outlined in the Declaration of Helsinki, written informed consent was not required, and patients were provided the option to opt out.

Between January 2013 and September 2019, a total of 736 patients diagnosed with CRC underwent curative surgical resection at the Department of Surgery and Oncology, Kyushu University Hospital. Patients who had received palliative procedures, such as stoma creation or partial resection without lymphadenectomy, those who had undergone combined organ resection, or those presenting with distant metastases were excluded from the study. As a result, 684 patients were included in the final analysis.

Clinicopathological data were retrospectively collected from medical records. The extracted data encompassed patient demographics (age, sex), comorbidities (e.g., diabetes mellitus, cardiac disease, cerebrovascular disease, respiratory disease), and perioperative parameters, including American Society of Anesthesiologists physical status (ASA-PS), preoperative albumin and C-reactive protein (CRP) levels, and preoperative leukocyte differential count. Additionally, pre- and postoperative levels of serum CEA (reference <5.0 ng/mL) and CA19-9 (reference <37.0 IU/mL) were recorded. Further data included information on preoperative chemotherapy and chemoradiotherapy, tumor location, surgical procedures performed, histological classification, TNM staging according to the Union for International Cancer Control (UICC) guidelines [14], the presence of lymphatic and vascular invasion, and postoperative complications categorized as Clavien-Dindo grade ≥2. Anastomotic leakage, survival outcomes, time to recurrence, and use of adjuvant chemotherapy were also documented. Details regarding the PET-CT protocol are described in a separate section. For evaluating nutritional and immunological status, the prognostic nutritional index (PNI) and the CRP-to-albumin ratio were assessed. Based on prior research [15], the cutoff values for these parameters were defined as 43.1 for PNI and 0.04 for the CRP-to-albumin ratio.

PET-CT image evaluation

The criteria for performing PET-CT at our department were as follows: (1) locally advanced cancer, (2) lymph node enlargement detected on other imaging modalities, and (3) elevated tumor markers. PET-CT was performed based not only on our department’s imaging criteria, but also as part of a diagnostic workup conducted by the internal medicine team after referral from our department.

PET-CT scans were conducted using either the Biograph mCT or Biograph Vision systems (Siemens Medical Solutions, Munich, Germany). Prior to imaging, patients fasted for a minimum of four hours. Fluorodeoxyglucose (FDG) was administered at a dose of 4 MBq per kilogram of body weight, followed by a 60-minute uptake period in a dim and quiet setting to optimize FDG distribution. The scanning range extended from the mid-thigh to the top of the head.

To quantify FDG uptake within the tumor, the standardized uptake value (SUV) was determined using non-contrast CT images, based on the following formula:

$\mathrm{SUV} = \frac{\text{Tissue activity (Bq/mL)} \times \text{Body weight (g)}}{\text{Injected activity (Bq)}}$

For each tumor, the SUVmax was defined as the highest FDG accumulation observed in any pixel. These measurements were obtained using the Fujifilm Synapse Vincent system (Fujifilm Corporation, Tokyo, Japan).

Statistical analysis

All statistical analyses were performed using JMP® software (version 17.0.0; SAS Institute, Cary, NC, USA). Comparisons of continuous variables between groups were conducted using the Mann-Whitney U test, while categorical variables were analyzed using the chi-square test or Fisher’s exact test. Spearman’s rank correlation coefficient tests were used to assess correlations between quantitative variables.

Receiver operating characteristic (ROC) curve analysis was employed to establish optimal cutoff values for predicting recurrence, based on sensitivity and specificity at various thresholds. Survival curves and median survival times were estimated using Kaplan-Meier analysis, with statistical differences assessed using the log-rank test. A Cox proportional hazards model was utilized to determine independent prognostic factors for overall survival (OS) and recurrence-free survival (RFS). A p-value of <0.05 was considered indicative of statistical significance.

## Results

Clinical and pathological characteristics of patients undergoing PET-CT

The clinical and pathological characteristics of the 164 patients who underwent PET-CT are summarized in Table 1. The cohort included both colon (50%) and rectal (50%) cancer cases, with a balanced sex distribution (80 females, 84 males), a median age of 67 years (range: 33-91), and a median BMI of 21.5 kg/m². Comorbidities were present in 45% of patients, and 10% had an ASA-PS ≥3. Elevated preoperative serum CEA and CA19-9 levels were observed in 35% and 13% of patients, respectively. No patients received neoadjuvant chemotherapy or chemoradiotherapy. According to UICC staging, 26% had stage 1, 37% stage 2, and 37% stage 3 disease. Lymph node metastasis was present in 37% of cases. Most tumors were well or moderately differentiated adenocarcinomas (91%), with the remainder being poorly differentiated or mucinous types. Vascular invasion and lymphatic invasion were observed in 32% and 16% of patients, respectively. Postoperative chemotherapy was administered to 34%, and complications occurred in 34%, including anastomotic leakage in 4% and transfusions in 7%. The recurrence rate was 16%.

**Table 1:** Clinicopathological characteristics of the patient who have undergone PET-CT imaging PET-CT: Positron Emission Tomography-Computed Tomography, BMI: body mass index, ASA-PS: American Society of Anesthesiologists physical status, CEA: carcinoembryonic antigen, CA19-9: carbohydrate antigen 19-9, PNI: prognostic nutritional index, CAR: C-reactive protein-to-albumin ratio, SUVmax: Standardized Uptake Value Max, CD: Clavien-Dindo, tub: tubular adenocarcinoma, por: poorly differentiated adenocarcinoma, muc: mucinous adenocarcinoma

Factor			PET-CT (+)
			n = 164
Sex	Female		80
	Male		84
Age (median, range)			67 (33–91)
BMI (median, range)			21.5 (14.9–33.0)
Comorbidities	Presence		74
	Absence		90
ASA-PS	1		19
	2		129
	3		16
Preoperative CEA (median, range)			3.5 (0.6–440.6)
Elevated preoperative CEA	≥ 5		58
Preoperative CA19-9 (median, range)			13.4 (0.4–484.7)
Elevated preoperative CA19-9	≥ 37		21
Postoperative CEA (median, range)			1.9 (0.3–1309)
Elevated postoperative CEA	≥ 5		26
Postoperative CA19-9 (median, range)			9.75 (0.6–303.6)
Elevated postoperative CA19-9	≥ 37		13
PNI (median, range)			46.0 (24.1–63.3)
CAR (median, range)			0.026 (0.002–2.548)
Preoperative therapy	Presence		0
	Absence		164
SUVmax of tumor (median, range)			11.5 (1.9–41.74)
SUVmax of lymph node (median, range)			2.99 (1.24–14.47)
Tumor location	Colon		81
	Rectum		83
Surgical procedures	Laparoscopic		149
	Open		15
Operative time (median, range)			329 (150–1008)
Estimated blood loss volume (median, range)			67 (1–3370)
Blood transfusion	Presence		11
	Absence		153
Histological type	Tub		149
	por + muc		15
Tumor diameter (median, range) (mm)			40 (0–130)
T factor	No residual tumor		0
	T1, 2		51
	T3, 4		113
Lymph node metastasis	Presence		61
	Absence		103
Pathological stage	No residual tumor		0
	1,2		103
	3		61
Lymphatic invasion	Presence		26
	Absence		138
Vascular invasion	Presence		52
	Absence		112
Postoperative complications (CD ≥ 2)	Presence		56
	Absence		108
Anastomotic leakage	Presence		7
	Absence		157
Postoperative chemotherapy	Presence		56
	Absence		108
Recurrence	Presence		26
	Absence		138

Accuracy of PET-CT in staging and lymph node metastasis detection

The accuracy of PET-CT in clinical staging and lymph node metastasis detection was assessed by comparing clinical and pathological staging results. When comparing clinical and pathological staging, the overall accuracy was 66.1% without PET-CT and 64.6% with PET-CT. Sensitivity for predicting pathological stage I, II, and III was 88.0%, 49.0%, and 55.6% without PET-CT, and 81.4%, 51.7%, and 65.6% with PET-CT, respectively. Cohen’s kappa coefficients were 0.483 and 0.465, indicating moderate agreement between clinical and pathological staging in both settings (Table 2).

**Table 2:** Discrepancy between preoperative clinical stage and pathological stage based on PET-CT status One patient with no residual tumor was excluded. PET-CT: Positron Emission Tomography-Computed Tomography

	Clinical stage				
Pathological stage	PET-CT (-)	Stage 1	Stage 2	Stage 3	Total
	Stage 1	176	15	9	200
	Stage 2	27	77	53	157
	Stage 3	43	29	90	162
	Total	246	121	152	519
	Clinical stage				
Pathological stage	PET-CT (+)	Stage 1	Stage 2	Stage 3	Total
	Stage 1	35	5	3	43
	Stage 2	5	31	24	60
	Stage 3	7	14	40	61
	Total	47	50	67	164

The diagnostic utility of PET-CT for lymph node metastasis was further evaluated. Among patients with pathological lymph node metastasis, PET-CT yielded a sensitivity of 57.4% and a positive predictive value (PPV) of 56.5%. Among those without metastasis, specificity was 73.8% and the negative predictive value (NPV) was 74.5%. The overall diagnostic accuracy was 67.7%. Chi-square test (p = 0.00014) and Fisher’s exact test (p = 0.00011) both demonstrated a significant association between PET-CT findings and pathological lymph node status (Table 3).

**Table 3:** Association between PET-CT lymph node uptake and final pathological lymph node metastasis PET-CT: Positron Emission Tomography-Computed Tomography, LN: lymph node

	PET-CT LN uptake			
LN metastasis		Presence	Absence	Total
	Presence	35	26	61
	Absence	27	76	103
	Total	62	102	164

Prognostic cutoff values of SUVmax and correlation between SUVmax and clinicopathological factors

**Figure 1:**
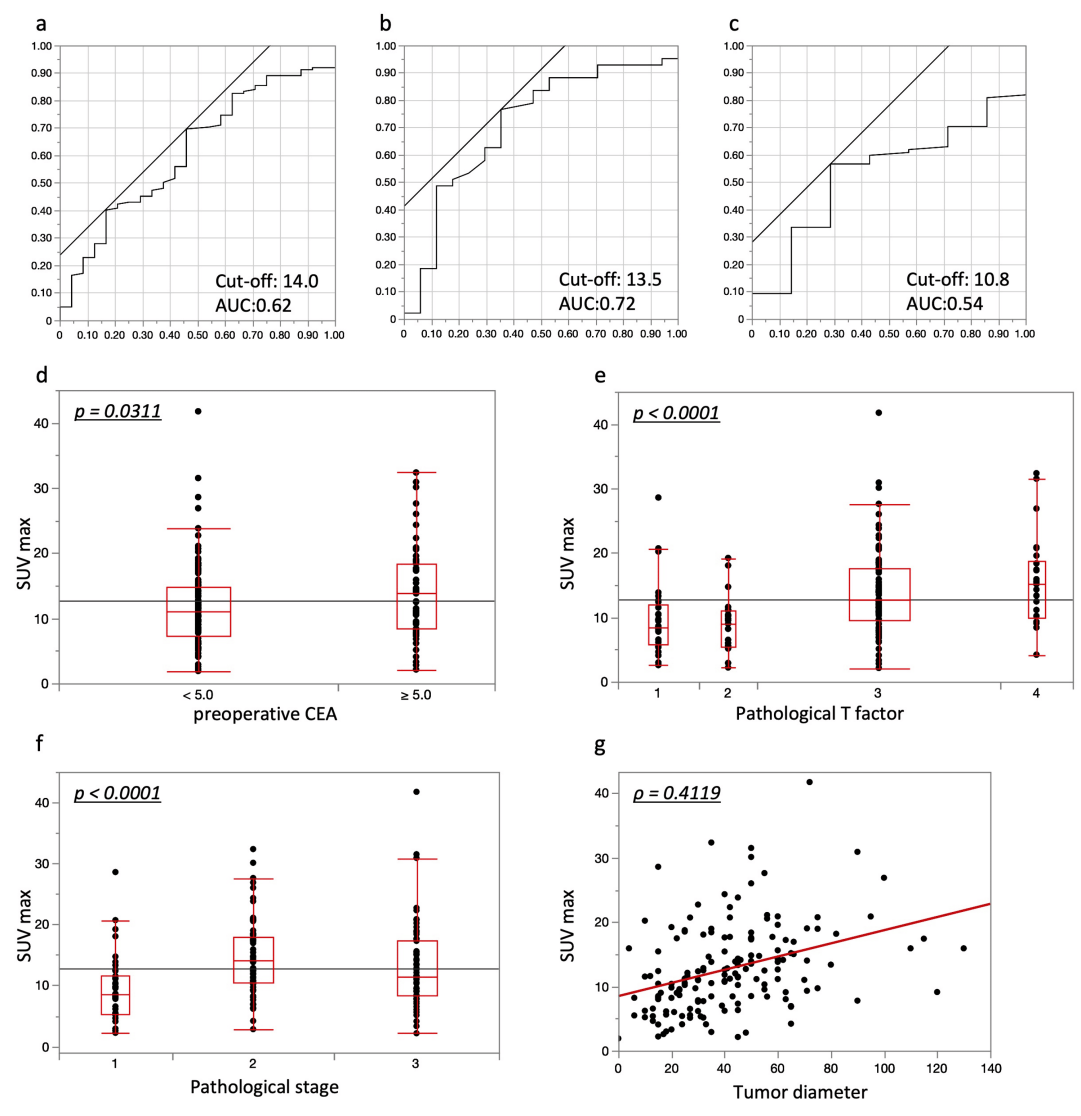
Prognostic cutoff values of SUVmax based on PET-CT. a. The overall SUVmax cutoff for recurrence prediction was 14.00 (AUC = 0.62), determined via ROC analysis. b. For Stage 3 patients, the cutoff was 13.5 (AUC = 0.72). c. For Stage 1 and 2 patients, the cutoff was 10.84 (AUC = 0.54). d–f. Correlation between SUVmax and clinicopathological factors: patients with elevated preoperative CEA levels, advanced T classification, and higher pathological stage had significantly higher SUVmax values (p = 0.0311, p < 0.0001, p < 0.0001, respectively). g. SUVmax was positively correlated with tumor diameter (ρ = 0.4119). SUVmax: Standardized Uptake Value Max, PET-CT: Positron Emission Tomography-Computed Tomography, CEA: carcinoembryonic antigen, ROC: eeceiver operating characteristic, AUC: area under the ROC curve

ROC curve analysis was used to determine the SUVmax cutoff values for recurrence prediction. In the PET-CT cohort, the overall cutoff value for SUVmax was 14.00 (area under the ROC curve (AUC) = 0.62), with a sensitivity of 70% and a specificity of 54% (Figure 1A). The stage-specific cutoff values were 13.5 for stage 3 (AUC = 0.72; sensitivity 77%, specificity 65%) (Figure 1B) and 10.84 for stages 1 and 2 (AUC = 0.54; sensitivity 57%, specificity 71%) (Figure 1C). The relationship between SUVmax and clinicopathological factors was examined. Patients with elevated preoperative CEA levels exhibited a significantly higher SUVmax than those with normal preoperative CEA levels (13.9 vs. 11.05, p = 0.0311). SUVmax values were also significantly higher in patients with more advanced T classifications (T1: 8.48, T2: 9.00, T3: 12.7, T4: 15.28, p < 0.0001) and pathological stages (Stage 1: 8.555, Stage 2: 14.15, Stage 3: 11.5, p < 0.0001) (Figure 1D-1F). Additionally, a significant positive correlation was observed between tumor diameter and SUVmax (ρ = 0.4119) (Figure 1G). There were no significant associations between SUVmax and other clinicopathological factors.

Clinicopathological factors associated with OS and RFS in patients undergoing PET-CT

The five-year OS and RFS rates were 87.1% and 84.3%, respectively. In multivariate analysis, advanced age (HR = 2.31, p = 0.0095) and anastomotic leakage (HR = 3.10, p = 0.033) were independent predictors of poorer OS. For RFS, advanced tumor stage (HR = 17.0, p = 0.0201), lymph node metastasis (HR = 21.7, p = 0.0096), and pathological stage (HR = 87.1, p < 0.001) were significant prognostic factors (Tables 4, 5).

**Table 4:** Clinicopathological factors associated with the overall survival of patients who have undergone PET-CT imaging *Indicates statistical significance HR: hazard ratio, CI: confidence interval, BMI: body mass index, ASA-PS: American Society of Anesthesiologists physical status, CEA: carcinoembryonic antigen, CA19-9: carbohydrate antigen 19-9, PNI: prognostic nutritional index, CAR: C-reactive protein-to-albumin ratio, SUVmax: Standardized Uptake Value Max, PET-CT: Positron Emission Tomography-Computed Tomography, CD: Clavien–Dindo, tub: tubular adenocarcinoma, por: poor differentiated adenocarcinoma, muc: mucinous adenocarcinoma

			Univariate		Multivariate	
Factor	Details	n	HR (95%CI)	p value	HR (95%CI)	p value
Age	≥ 75	43	2.31 (1.21–4.40)	0.0110*	2.35 (1.23–4.48)	0.0095*
Sex	Male	84	1.76 (0.91–3.41)	0.0918		
BMI	< 25	134	1.84 (0.72–4.72)	0.2052		
Comorbidities	Presence	74	1.69 (0.89–3.23)	0.1095		
ASA-PS	≥ 3	16	2.15 (0.95–4.89)	0.0675		
Preoperative CEA	≥ 5	59	1.80 (0.95–3.41)	0.0702		
Preoperative CA19-9	≥ 37	23	1.67 (0.73–3.79)	0.2221		
Postoperative CEA	≥ 5	16	2.02 (0.84–4.84)	0.1157		
Postoperative CA19-9	≥ 37	11	1.82 (0.65–5.14)	0.2576		
PNI	< 43.1	47	1.81 (0.95–3.48)	0.0729		
CAR	≥ 0.04	62	1.78 (0.94–3.38)	0.0755		
Tumor location	Rectum	85	1.16 (0.61–2.21)	0.6407		
SUVmax of tumor	≥ 14.00	59	1.22 (0.65–2.31)	0.5359		
Lymph node uptake on PET-CT	Presence	62	1.45 (0.76–2.76)	0.2552		
Operative time	≥ 300	96	1.31 (0.69–2.47)	0.4087		
Estimated blood loss volume	≥ 100	64	1.19 (0.62–2.31)	0.5996		
Blood transfusion	Presence	11	2.16 (0.30–15.83)	0.448		
Histological type	por, muc	15	1.70 (0.67–4.36)	0.2705		
Tumor size	≥ 50	56	1.43 (0.75–2.72)	0.2779		
Tumor stage	T3, T4	113	1.31 (0.63–2.69)	0.4707		
Lymph node metastasis	Presence	61	1.17 (0.61–2.27)	0.6335		
Pathological stage	3	61	1.12 (0.59–2.16)	0.7243		
Lymphatic invasion	Presence	26	1.20 (0.50–2.88)	0.6789		
Vascular invasion	Presence	52	1.11 (0.57–2.17)	0.7594		
Postoperative complications (CD ≥ 2)	Presence	56	1.74 (0.92–3.30)	0.0903		
Anastomotic leakage	Presence	7	2.96 (1.05–8.38)	0.0404*	3.10 (1.10–8.77)	0.0329*
Postoperative adjuvant chemotherapy	Presence	108	1.03 (0.53–1.99)	0.9321		

**Table 5:** Clinicopathological factors associated with the recurrence-free survival of patients who have undergone PET-CT imaging *Indicates statistical significance HR: hazard ratio, CI: confidence interval, BMI: body mass index, ASA-PS: American Society of Anesthesiologists physical status, CEA: carcinoembryonic antigen, CA19-9: carbohydrate antigen 19-9, PNI: prognostic nutritional index, CAR: C-reactive protein-to-albumin ratio, SUVmax: Standardized Uptake Value Max, PET-CT: Positron Emission Tomography-Computed Tomography, CD: Clavien–Dindo, tub: tubular adenocarcinoma, por: poor differentiated adenocarcinoma, muc: mucinous adenocarcinoma

			Univariate		Multivariate	
Factor	Details	n	HR (95%CI)	p value	HR (95%CI)	p value
Age	≥ 75	43	1.21 (0.48–3.01)	0.6852		
Sex	Male	84	1.35 (0.62–2.96)	0.4418		
BMI	< 25	134	3.05 (0.72–12.89)	0.1304		
Comorbidities	Presence	74	1.29 (0.58–2.84)	0.5319		
ASA-PS	≥ 3	16	2.62 (0.99–6.96)	0.0529		
Preoperative CEA	≥ 5	59	1.94 (0.90–4.19)	0.0915		
Preoperative CA19-9	≥ 37	23	2.04 (0.82–5.08)	0.126		
Postoperative CEA	≥ 5	16	3.02 (1.21–7.54)	0.0175*	1.70 (0.61–4.75)	0.3109
Postoperative CA19-9	≥ 37	11	2.86 (0.99–8.32)	0.0531		
PNI	< 43.1	47	1.16 (0.51–2.67)	0.7227		
CAR	≥ 0.04	62	1.11 (0.50–2.44)	0.7995		
Tumor location	Rectum	85	1.10 (0.51–2.37)	0.8131		
SUVmax of tumor	≥ 14.00	59	1.12 (0.51–2.46)	0.7852		
Lymph node uptake on PET-CT	Presence	62	2.52 (1.16–5.49)	0.0199*	1.29 (0.56–2.95)	0.552
Operative time	≥ 300	96	1.59 (0.69–3.66)	0.2748		
Estimated blood loss volume	≥ 100	64	1.56 (0.73–3.39)	0.2507		
Blood transfusion	Presence	11	1.79 (0.24–13.20)	0.5675		
Histological type	por, muc	15	1.49 (0.45–4.95)	0.5194		
Tumor size	≥ 50	56	1.08 (0.48–2.41)	0.8588		
Tumor stage	≥ 3	113	12.63 (1.71–93.26)	0.0129*	17.02 (1.56–185.90)	0.0201*
Lymph node metastasis	Presence	61	4.05 (1.76–9.32)	0.0010*	21.69 (2.11–222.54)	0.0096*
Pathological stage	3	61	5.80 (2.33–14.46)	0.0002*	87.11 (9.79–774.94)	< 0.0001*
Lymphatic invasion	Presence	26	1.81 (0.73–4.51)	0.202		
Vascular invasion	Presence	52	2.21 (1.02–4.77)	0.0431*	1.40 (0.60–3.28)	0.4359
Postoperative complications (CD ≥ 2)	Presence	56	1.30 (0.59–2.87)	0.5106		
Anastomotic leakage	Presence	7	6.46 (2.21–18.86)	0.0006*	3.26 (0.93–11.45)	0.0655
Postoperative adjuvant chemotherapy	Presence	108	2.74 (1.26–5.96)	0.0112*	1.35 (0.48–3.80)	0.5739

Prognostic impact of SUVmax in stage 3 CRC

In stage 3 patients, high SUVmax was independently associated with worse OS (HR = 4.44, p = 0.049), along with anastomotic leakage (HR = 78.3, p = 0.028). RFS was significantly affected by high SUVmax (HR = 3.47, p = 0.020), ASA-PS ≥3 (HR = 8.39, p = 0.005), and high blood loss (HR = 3.00, p = 0.041) (Tables 6, 7).

**Table 6:** Analysis of clinicopathological factors associated with overall survival in stage 3 colorectal cancer patients undergoing PET-CT *Indicates statistical significance HR: hazard ratio, CI: confidence interval, BMI: body mass index, ASA-PS: American Society of Anesthesiologists physical status, CEA: carcinoembryonic antigen, CA19-9: carbohydrate antigen 19-9, PNI: prognostic nutritional index, CAR: C-reactive protein-to-albumin ratio, SUVmax: Standardized Uptake Value Max, PET-CT: Positron Emission Tomography-Computed Tomography, CD: Clavien–Dindo, por: poor differentiated adenocarcinoma, muc: mucinous adenocarcinoma

			Univariate		Multivariate	
Factor	Details	n	HR (95%CI)	p value	HR (95%CI)	p value
Age	≥ 75	11	2.39 (0.80–7.16)	0.1191		
Sex	Male	29	2.26 (0.76–6.75)	0.1451		
BMI	< 25	48	4.38 (0.57–33.6)	0.1546		
Comorbidities	Presence	22	1.43 (0.50–4.13)	0.5071		
ASA-PS	≥ 3	4	5.57 (1.54–20.1)	0.0087*	2.85 (0.58–13.9)	0.1948
Preoperative CEA	≥ 5	25	3.04 (1.02–9.10)	0.0465*	2.10 (0.58–7.61)	0.2596
Preoperative CA19-9	≥ 37	10	2.87 (0.89–9.23)	0.0763		
Postoperative CEA	≥ 5	8	3.44 (1.06–11.1)	0.0395*	1.53 (0.15–16.1)	0.7211
Postoperative CA19-9	≥ 37	4	1.08 (0.14–8.30)	0.943		
PNI	< 43.1	18	2.28 (0.79–6.59)	0.1281		
CAR	≥ 0.04	20	2.17 (0.74–6.32)	0.1566		
Tumor location	Rectum	32	2.22 (0.72–6.84)	0.1662		
SUVmax of tumor	≥ 13.5	22	3.77 (1.26–11.3)	0.0179*	4.31 (1.04–18.0)	0.0444*
Lymph node uptake on PET-CT	Presence	35	1.75 (0.58–5.30)	0.3191		
Operative time	≥ 300	37	3.20 (0.89–11.5)	0.0752		
Estimated blood loss volume	≥ 100	26	2.73 (0.91–8.17)	0.0725		
Blood transfusion	Presence	1	14.3 (1.60–127.9)	0.0174*	2.64 (0.16–44.0)	0.4999
Histological type	por, muc	9	1.20 (0.27–5.37)	0.8133		
Tumor size	≥ 50	24	1.31 (0.46–3.78)	0.6151		
Tumor stage	T3, T4	53	2.52 (0.33–19.3)	0.3742		
Lymphatic invasion	Presence	17	1.31 (0.41–4.81)	0.6528		
Vascular invasion	Presence	33	1.10 (0.38–3.19)	0.8534		
Postoperative complications (CD ≥ 2)	Presence	22	2.51 (0.87–7.22)	0.0885		
Anastomotic leakage	Presence	3	2.96 (1.05–8.38)	< 0.0001*	31.2 (1.62–601.6)	0.0226*
Postoperative adjuvant chemotherapy	Absence	15	1.41 (0.44–4.49)	0.5644		

**Table 7:** Analysis of clinicopathological factors associated with recurrence-free survival in stage 3 colorectal cancer patients undergoing PET-CT *Indicates statistical significance HR: hazard ratio, CI: confidence interval, BMI: body mass index, ASA-PS: American Society of Anesthesiologists physical status, CEA: carcinoembryonic antigen, CA19-9: carbohydrate antigen 19-9, PNI: prognostic nutritional index, CAR: C-reactive protein-to-albumin ratio, SUVmax: Standardized Uptake Value Max, PET-CT: Positron Emission Tomography-Computed Tomography, CD: Clavien–Dindo, por: poor differentiated adenocarcinoma, muc: mucinous adenocarcinoma

			Univariate		Multivariate	
Factor	Details	n	HR (95%CI)	p value	HR (95%CI)	p value
Age	≥ 75	11	1.46 (0.48–4.48)	0.5081		
Sex	Male	29	1.24 (0.48–3.22)	0.6568		
BMI	< 25	48	5.25 (0.70–39.6)	0.1077		
Comorbidities	Presence	22	1.87 (0.72–4.86)	0.1999		
ASA-PS	≥ 3	4	6.03 (1.66–21.9)	0.0062*	8.39 (1.91–37.0)	0.0049*
Preoperative CEA	≥ 5	25	1.44 (0.55–3.73)	0.4554		
Preoperative CA19-9	≥ 37	10	1.36 (0.31–5.93)	0.6858		
Postoperative CEA	≥ 5	8	1.58 (0.46–5.52)	0.4693		
Postoperative CA19-9	≥ 37	4	1.81 (0.15–4.30)	0.9992		
PNI	< 43.1	18	1.51 (0.56–4.09)	0.4199		
CAR	≥ 0.04	20	1.35 (0.50–3.65)	0.5587		
Tumor location	Rectum	32	1.72 (0.63–4.65)	0.2871		
SUVmax of tumor	≥ 13.5	22	4.19 (1.55–11.4)	0.0049*	3.47 (1.22–9.93)	0.0201*
Lymph node uptake on PET-CT	Presence	35	2.14 (0.75–6.08)	0.1536		
Operative time	≥ 300	37	1.70 (0.60–4.84)	0.302		
Estimated blood loss volume	≥ 100	26	2.78 (1.02–7.53)	0.0440*	3.00 (1.04–8.65)	0.0413*
Blood transfusion	Presence	1	29.5 (2.67–325.3)	0.0057*	4.21 (0.20–88.3)	0.3544
Histological type	por, muc	9	1.14 (0.26–4.99)	0.8619		
Tumor size	≥ 50	24	1.18 (0.45–3.12)	0.7279		
Tumor stage	T3, T4	53	2.94 (0.39–22.2)	0.2954		
Lymphatic invasion	Presence	17	1.25 (0.44–3.55)	0.6773		
Vascular invasion	Presence	33	1.15 (0.44–3.02)	0.7779		
Postoperative complications (CD ≥ 2)	Presence	22	1.43 (0.54–3.77)	0.4667		
Anastomotic leakage	Presence	3	5.56 (1.22–25.2)	0.0264*	4.96 (0.57–43.4)	0.1478
Postoperative adjuvant chemotherapy	Absence	15	1.60 (0.56–4.56)	0.3759		

Prognostic impact of SUVmax and tumor markers on OS and RFS

**Figure 2:**
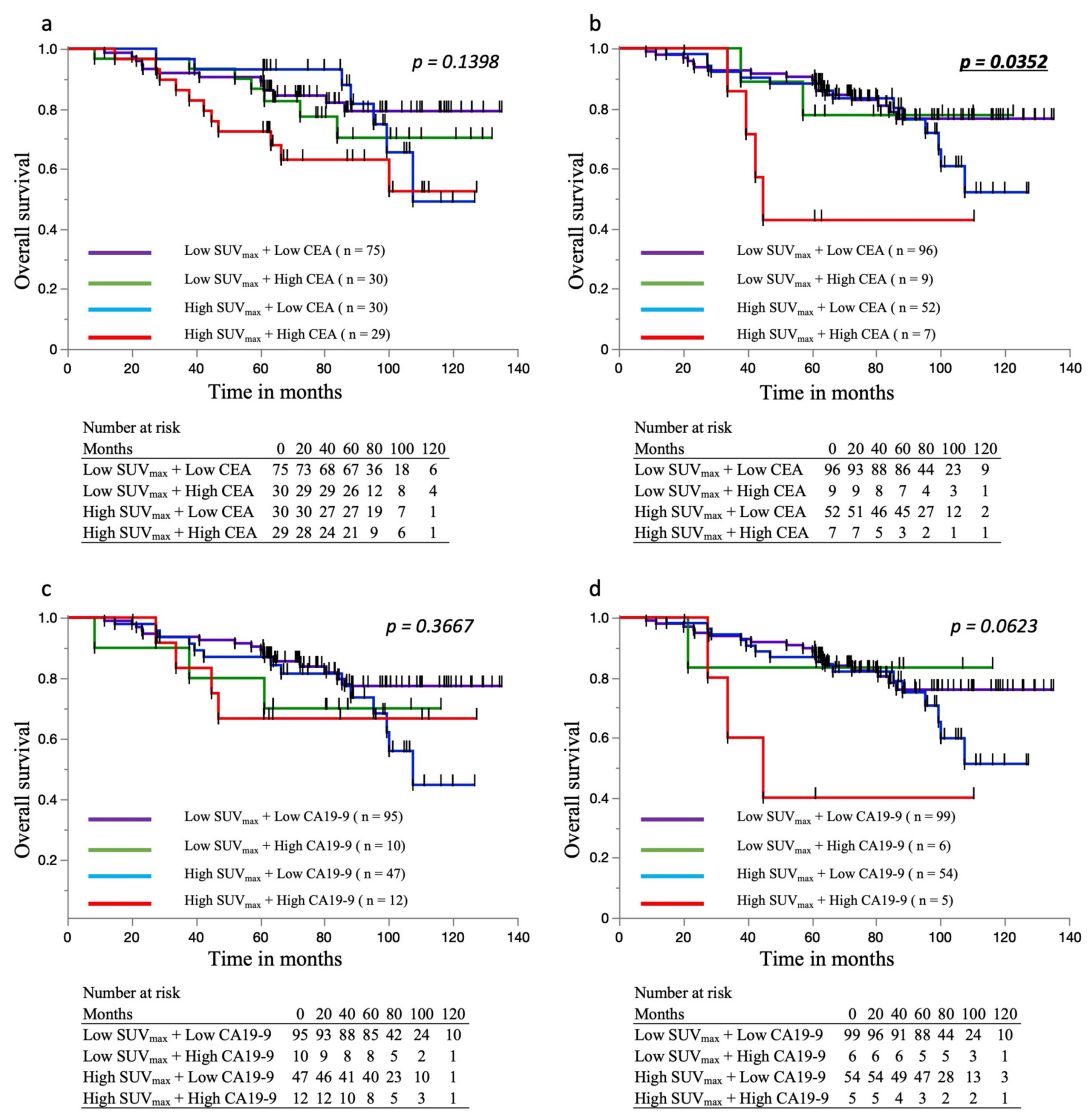
Overall survival rates based on SUVmax and pre/postoperative tumor markers a. OS stratified by preoperative CEA and SUVmax. No significant difference was observed. b. OS stratified by postoperative CEA and SUVmax. The High SUVmax + High CEA group showed the worst survival, with a significant difference. c. OS stratified by preoperative CA19-9 and SUVmax. No significant difference was observed. d. OS stratified by postoperative CA19-9 and SUVmax. The High SUVmax + High CA19-9 group had the worst survival, though not statistically significant. OS: overall survival, SUVmax: Standardized Uptake Value Max, CEA: carcinoembryonic antigen, CA19-9: carbohydrate antigen 19-9

**Figure 3:**
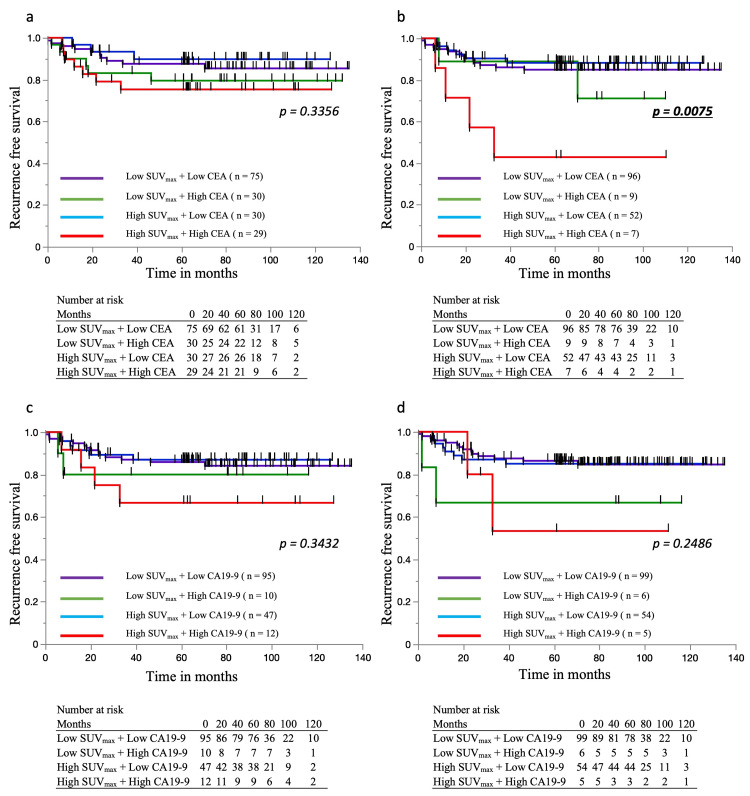
Recurrence-free survival rates based on SUVmax and pre/postoperative tumor markers. a. RFS stratified by preoperative CEA and SUVmax. No significant difference was observed. b. RFS stratified by postoperative CEA and SUVmax. The High SUVmax + High CEA group showed the worst RFS, with a significant difference. c. RFS stratified by preoperative CA19-9 and SUVmax. No significant difference was observed. d. RFS stratified by postoperative CA19-9 and SUVmax. The High SUVmax + High CA19-9 group had the worst RFS, though not statistically significant. RFS: recurrence-free survival, SUVmax: Standardized Uptake Value Max, CEA: carcinoembryonic antigen, CA19-9: carbohydrate antigen 19-9

Patients were categorized into four groups based on SUVmax (≥14 or <14) and pre-/postoperative tumor markers (CEA, CA19-9), as shown in Figure 2 and Figure 3. For OS, no significant differences were observed in the preoperative CEA and CA19-9 classifications (Figure 2A-2C). However, postoperative CEA classification showed significantly worse survival in the high SUVmax + high CEA group (p = 0.035; Figure 2B), and a similar trend was noted for postoperative CA19-9 (Figure 2D). For RFS, preoperative classifications again showed no significant differences (Figure 3A, 3C), while postoperative CEA was a significant prognostic factor (p = 0.007; Figure 3B). Postoperative CA19-9 showed a non-significant trend toward poorer RFS in the high SUVmax + high marker group (Figure 3D).

## Discussion

This study evaluated the clinical utility of PET-CT for preoperative staging and prognostic assessment in resectable CRC patients. The key findings were as follows: (1) Despite PET-CT use, preoperative staging accuracy remained suboptimal, and its ability to detect lymph node metastasis was limited. (2) High SUVmax alone had limited prognostic value; however, when combined with elevated tumor markers such as CEA, it was more strongly associated with worse OS and RFS. (3) Postoperative tumor markers, particularly CEA, in combination with high SUVmax were predictive of recurrence, with higher values indicating an increased risk. These findings suggest that PET-CT alone is insufficient for accurate staging and prognosis in resectable CRC. However, when integrated with tumor markers like CEA, its prognostic utility improves, allowing for better risk stratification. To our knowledge, this study is among the first to comprehensively evaluate the prognostic impact of SUVmax in conjunction with tumor markers and clinicopathological factors in resectable CRC patients undergoing PET-CT. Our results highlight the potential role of this combined approach in refining preoperative assessment.

Our findings align with previous research on CRC prognostic factors. A prior study demonstrated that osteosarcopenia serves as a predictor of postoperative complications and recurrence risk, underscoring the importance of patient-specific biological markers in prognostic evaluation [12]. Similarly, previous studies have indicated that single-factor prognostic indicators, such as muscle mass indices and inflammatory markers, have limited predictive value when used in isolation [15,16]. Given these limitations, an integrative prognostic model incorporating PET-CT findings, metabolic parameters, and systemic inflammation markers may enhance predictive accuracy [17,18]. 

SUVmax was significantly associated with elevated preoperative CEA levels, advanced T classification, and pathological stage in CRC. These findings suggest that SUVmax reflects not only tumor burden and metabolic activity but also involvement in cellular metabolic reprogramming. This view is supported by translational research, including a review by Jadvar et al. [19], which highlights how increased 18F-FDG uptake is associated with molecular features of aggressive tumor behavior in colorectal cancer. This supports the biological relevance of SUVmax as an indicator of tumor invasiveness. Increased FDG uptake has been attributed to the overexpression of glucose transporter 1 and the activation of hexokinase-2, both of which are regulated by hypoxia-inducible factor-1 alpha and the PI3K/AKT/mTOR signaling pathway. These molecular alterations are known to be activated in highly invasive CRC [20]. Additionally, SUVmax is influenced not only by the metabolic activity of the tumor itself but also by the tumor microenvironment [21], and may serve as an indicator of tumor aggressiveness and metabolic adaptation. Although our study did not include pre- and post-treatment SUVmax comparisons due to the absence of neoadjuvant therapy, previous reports have shown that changes in SUVmax before and after treatment are associated with prognosis [8]. These findings suggest a possible correlation between SUVmax and chemotherapy response. However, further studies are required to determine whether high SUVmax directly correlates with poor prognosis and recurrence in CRC.

KRAS mutations in CRC are associated with constitutive activation of the MAPK pathway, which has been reported to enhance glycolysis and subsequently increase FDG uptake [22]. Similarly, BRAF-mutant tumors tend to exhibit higher malignancy and may demonstrate elevated SUVmax. Our analysis revealed a trend toward higher SUVmax in KRAS-mutant cases compared to KRAS wild-type cases (KRAS-mutant: 13.26, n = 16 vs. KRAS wild-type: 10.35, n = 10, p = 0.1076; data not shown); however, the small sample size limits definitive conclusions. Further case accumulation is necessary for a more comprehensive evaluation.

Another important consideration is the role of PET-CT in detecting micrometastatic disease that may not be apparent with conventional imaging techniques. Although this study did not identify a strong association between SUVmax and recurrence risk in early-stage CRC, PET-CT may still be useful in a subset of high-risk patients. For example, patients with high-risk pathological features such as lymphovascular invasion, perineural invasion, or elevated inflammatory markers might benefit from advanced imaging for improved recurrence risk stratification [23,24]. Future research should investigate whether integrating PET-CT findings with molecular and genetic markers, such as circulating tumor DNA (ctDNA) [25], could improve predictive accuracy. In particular, evaluating SUVmax in patients with positive ctDNA status after surgery may help identify those at higher risk of recurrence and guide the selection of appropriate adjuvant chemotherapy regimens.

This study also raises questions regarding the metabolic activity of CRC and its implications for treatment strategies. While SUVmax reflects tumor glucose metabolism, other metabolic pathways, such as lipid and amino acid metabolism, may also influence tumor progression and recurrence. Recent advances in metabolomics and radiomics suggest the potential of alternative imaging biomarkers beyond FDG-PET [26,27]. Exploring the integration of these approaches may provide a more comprehensive understanding of tumor biology and refine patient selection for PET-CT-based risk assessment.

Given the high costs associated with PET-CT, its clinical utility must be weighed against cost-effectiveness. Our findings suggest that routine PET-CT use in all resectable CRC patients may not be justified, particularly in early-stage disease, where its predictive value is limited. This aligns with previous research on CRC prognosis, which underscores the need for cost-efficient and clinically meaningful predictive tools [28]. Healthcare resources could be better allocated to alternative, more cost-effective recurrence risk assessment methods, such as serum tumor markers (CEA and CA19-9) or novel imaging techniques with greater prognostic accuracy [29]. Future studies should conduct cost-benefit analyses to further evaluate the financial implications of routine PET-CT use in CRC patients, particularly in the context of radiomics and artificial intelligence-based predictive models.

This study has several limitations. First, as a retrospective analysis, selection bias cannot be excluded. While PET-CT was primarily performed in patients with locally advanced disease, suspected lymph node metastasis, or elevated tumor markers, some patients underwent PET-CT as part of evaluations for other diseases at external institutions or as part of internal medicine assessments. This variation in PET-CT indications may have introduced selection bias; however, the inclusion of patients who underwent PET-CT in a more random manner may have mitigated this effect to some extent. Second, the study population was derived from a single institution, which may limit the generalizability of the findings. Third, the sample size of patients undergoing PET-CT was relatively small compared to the overall cohort, potentially affecting the statistical power of the analysis. In addition, the multivariate analysis is subject to statistical limitations due to the relatively small number of events in relation to the number of variables included. As a result, the model may be at risk of overfitting, and the results should be interpreted with caution. Furthermore, multiple subgroup analyses were performed without formal correction for multiple testing, as this was an exploratory evaluation. Therefore, the observed associations should also be interpreted with care due to the potential risk of type I error. SUVmax was the primary PET-CT parameter evaluated, whereas other metabolic parameters, such as metabolic tumor volume (MTV) and total lesion glycolysis (TLG), were not assessed, despite their potential prognostic value. Future prospective, multicenter studies incorporating a broader range of imaging biomarkers are needed to validate these findings and refine recurrence prediction models for CRC patients.

## Conclusions

In conclusion, SUVmax on preoperative PET-CT demonstrated prognostic value for recurrence and survival in stage 3 colorectal cancer, especially when combined with postoperative CEA levels. This combination identified a distinct subgroup of patients with particularly poor outcomes, highlighting the potential of SUVmax as a surrogate marker of tumor burden and biological aggressiveness. However, PET-CT showed limited utility in early-stage disease and only moderate accuracy for lymph node staging. These findings suggest that routine use of PET-CT in all CRC patients is not warranted. Instead, a more selective approach - targeting patients with high-risk features such as advanced stage, elevated tumor markers, or equivocal CT findings - may optimize clinical decision-making and resource allocation. Further prospective studies are needed to validate these findings and explore the integration of PET-CT with other prognostic tools to improve individualized treatment planning in colorectal cancer care.
